# Identification of growth years for Puerariae Thomsonii Radix based on hyperspectral imaging technology and deep learning algorithm

**DOI:** 10.1038/s41598-023-40863-6

**Published:** 2023-08-31

**Authors:** Lei Zhang, Yu Guan, Ni Wang, Fei Ge, Yan Zhang, Yuping Zhao

**Affiliations:** 1 China Academy of Chinese Medical Sciences, No.16, Nanxiao Street, Dongzhimen, Dongcheng District, Beijing, 100700 People’s Republic of China; 2 School of Pharmacy, Jiangxi University of Chinese Medicine, Nanchang, 300004 People’s Republic of China; 3 GAP Center, Heilongjiang University of Chinese Medicine, Harbin, 150040 People’s Republic of China; 4 School of Materials Science and Engineering, Zhejiang University, No.866, Yuhangtang, Xihu District, Hangzhou, 310058 People’s Republic of China

**Keywords:** Plant sciences, Drug discovery

## Abstract

Puerariae Thomsonii Radix (PTR) is not only widely used in disease prevention and treatment but is also an important raw material as a source of starch and other food. The growth years of PTR are closely related to its quality. The rapid and nondestructive identification of growth year is essential for the quality control of PTR and other traditional Chinese medicines. In this study, we proposed a convolutional neural network (CNN)-based classification framework in conjunction with hyperspectral imaging (HSI) technology for the rapid identification of the growth years of PTRs. Traditional treatment methods (i.e., multiplicative scatter correction, standard normal variate, and Savitzky-Golay smoothing) combined with machine learning algorithms (i.e., random forest, logistic regression, naive Bayes, and eXtreme gradient boost) were used as baseline models. Among them, the F1-score of CNN-based models based on PTRs’ outer surfaces was over 90%, outperforming all the other baseline models. These results showed that it was feasible to use a deep learning algorithm in conjunction with HSI technology to identify the growth years of PTR. This method provides a fast, nondestructive, and simple method of identifying the growth years of PTR. It can be easily applied to other scenarios, such as for the identification of the locality or years of growth for other traditional Chinese herbs.

## Introduction

*Pueraria Thomsonii* Benth (PTB) is a type of perennial vine, its root is included in the *Chinese Pharmacopoeia* which is named Puerariae Thomsonii Radix (PTR). PTRs are enriched with a variety of chemical components such as isoflavones, terpenoids, coumarins. PTRs have long been used as a type of traditional Chinese medicine. They have an apparent therapeutic effect and have been shown to improve cardiovascular diseases, anti-inflammation and analgesia, have an anti-diabetic effect, reduce the effects of alcohol, protect the liver, lighten skin, enlarge breasts^1–6^. They are also a type of healthy and famous food in China and Southeast Asia. PTRs have high economic value and market demand.

According to the *Chinese Pharmacopoeia*, the puerarin content (C_21_H_20_O_9_) affects PTR's quality and medicinal value. The higher the puerarin content in a PTR, the higher the quality of the PTR. PTRs with different growing ages vary in puerarin content. The puerarin content in PTR is directly related to the number of growth years. Xiong et al.^7^ found that 1-year-old PTB has a low puerarin level that is far below the pharmacopeia standards, meaning it can only be used as food or as a raw material. In contrast, PTB aged two years or more usually reaches the standard puerarin content level and can be used in traditional Chinese medicine. These findings indicate the importance of identifying the growth years of PTRs because growth years are directly related to PTRs' quality and economical and medicinal value.

The growth year of PTR is usually identified according to objective experience or using physical and chemical testing. However, PTRs with different growing ages have similar appearances; thus, it is difficult to distinguish their characteristics and colors based on objective judgment. In the past, the chemical identification of PTR has mainly been performed using High-Performance Liquid Chromatography (HPLC)^8,9^, which is time-consuming, laborious, costly, and destructive. Therefore, it seems that the two abovementioned methods cannot be used to identify the growing years of PTRs with high accuracy and efficiency and cannot satisfy the needs of industrial production.

Compared with traditional spectral technology, hyperspectral imaging (HSI) technology can be used to simultaneously collect surface image information and spectral information from a tested sample. Many researchers have used HSI to identify growing years and control the quality of traditional Chinese medicines. In the past few years, the accuracy of the identification of growth years for Glycyrrhizae Radix et Rhizoma^10^, Ophiopogonis Radix^11^, Ziziphi Spinosae Semen^12^, and Atractylodis Rhizoma^13^ reached 97.53%, 99.1%, 99.14%, and 97.3%, respectively. Zheng et al.^14^ explored the authentication of Armeniacae Semen Amarum and Persicae Semen based on HSI technology. Based on the comparative analysis of several different pre-processing methods and identification models, the researchers found that the second derivative pre-processing model and partial least squares discriminant analysis were the best model combination. The accuracy of classification reached 100%. Cheng et al.^15^ screened 20 characteristic wavelengths using the successive projections algorithm and established several models to identify the origin of Frankincense. The results showed that the accuracies of the extreme learning machine and linear discriminant analysis were 100%. To the best of our knowledge, no reports have focused on the application of HSI technology in the identification of growth years of PTR. Deep learning methods such as conventional neural networks (CNNs) have been widely used in many fields, such as image classification^16^, content prediction^17^, etc., showing high performance and good generalization. In this study, we proposed a CNN-based classification framework to identify growth years of PTRs based on hyperspectral images. Here, traditional treatment methods (i.e., multiplicative scatter correction (MSC), standard normal variate (SNV), and Savitzky–Golay smoothing (SG)) in conjunction with several state-of-the-art machine learning models were used as baseline methods to demonstrate the effectiveness and superiority of the proposed method.

## Materials and methods

### Hyperspectral imaging system

**Figure 1:**
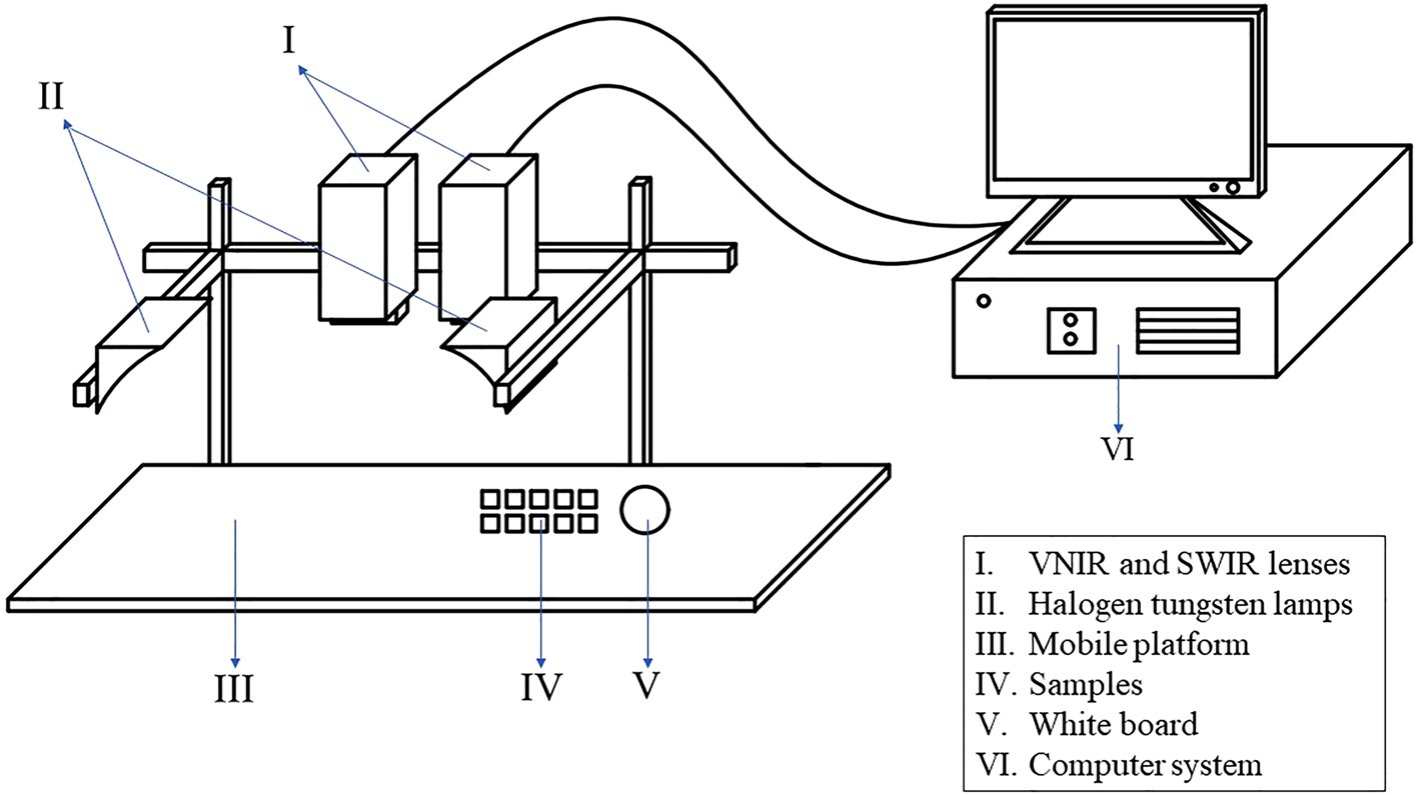
Hyperspectral imaging system. *VNIR* visible and near-infrared lens, *SWIR* short-wave infrared lens. HSI system consists mainly of lenses, light sources, mobile platform, and computer system.

The HSI system used in this study was the HySpex series produced by Norsk Elektro Optikk AS (Norway). The system consists of two lenses, two halogen tungsten lamps, a CCD detector, a mobile platform, and its supporting computer system and software (Fig. 1). The two lenses in the instrument are a visible and near-infrared lens (VNIR) SN0605 VNIR (spectral range 410–990 nm) and a short-wave infrared lens (SWIR) N3124 SWIR (spectral range 950–2500 nm). The VNIR lens has a total of 108 bands, and the SWIR lens has 288 bands. The two lenses are vertically fixed on a bracket 30 cm away from the moving platform. The moving speed of the platform is 1.5 mm/s. The angle between the light source and the platform is 45°. The camera can be connected to a computer via a cable to obtain hyperspectral images. The integration time and frame period of the VNIR lens and SWIR lens are 9000 μs and 3500 μs; and 41,501 μs and 108,199 μs, respectively.

The quality of scanning images might be affected by a dark current in the lens and noise caused by an uneven light source distribution. Therefore, a clean, standard-reflectivity whiteboard was added behind the sample as a reference. Then, a blackboard reference image was obtained in all-black mode. The original hyperspectral image was corrected with the black-and-white reference image obtained^18,19^. The calibration formula is defined as follows:

1 $$R=\frac{{R}_{0}-{R}_{b}}{{R}_{w}-{R}_{b}},$$

where *R* is the calibrated reflectance image, *R*_0_ is the raw reflectance image, *R*_w_ is the white reference image, and *R*_b_ is the dark reference image. Before imaging, we repeatedly tested and adjusted the parameters of the HSI system to ensure the exposure degree and reduce noise, wherein the lens height and illumination position were fixed at 30 cm and 45°, respectively.

### Sample

**Figure 2:**
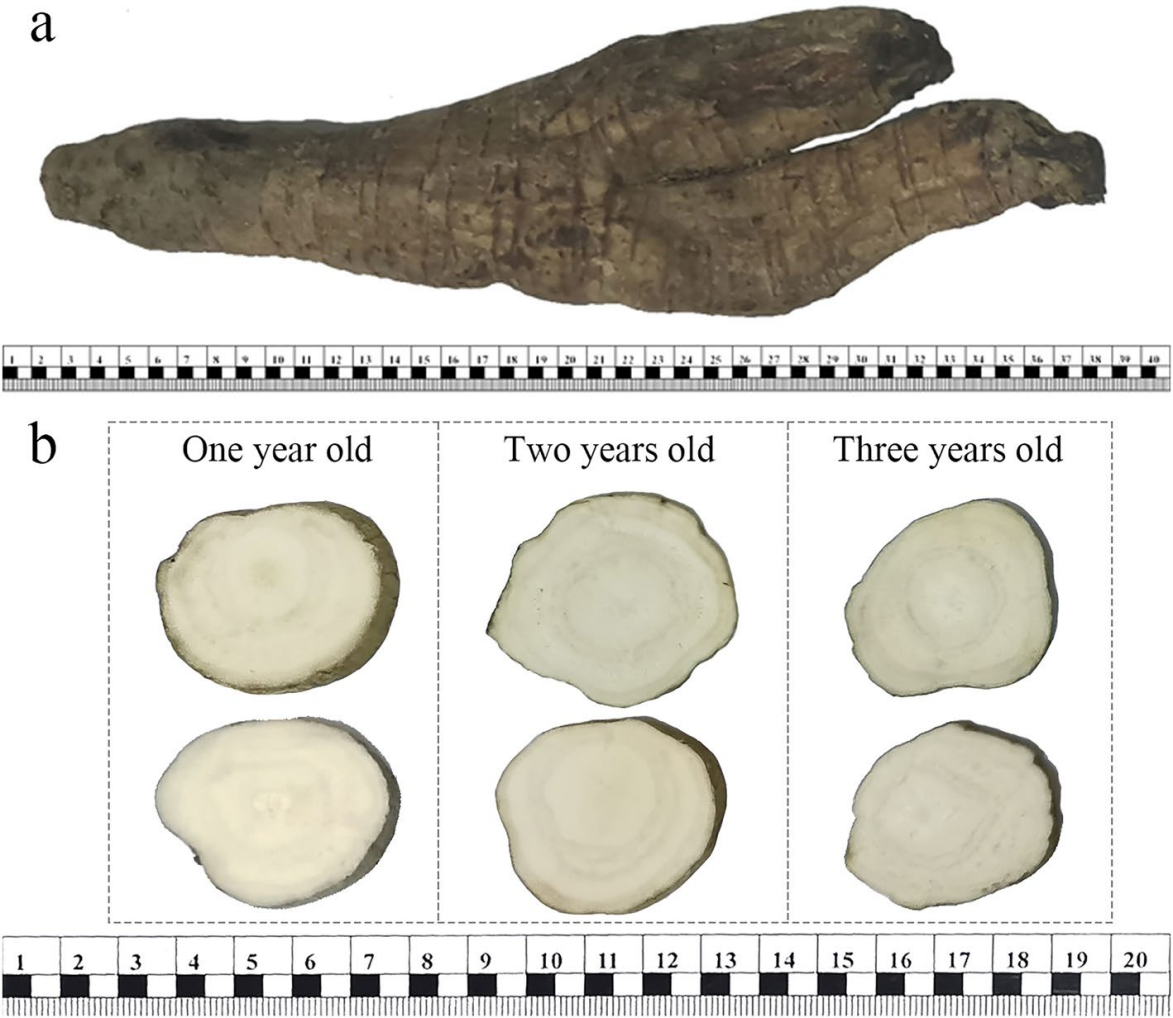
Preparation of Puerariae Thomsonii Radix samples. (**a**) Outer surface of Puerariae Thomsonii Radix samples; (**b**) cross-sections of Puerariae Thomsonii Radix samples.

Inflated root tubers of the cultivated PTRs were collected in April 2021. A total of 75 healthy PTRs with different growth years were collected at the Puerariae Cultivation Demonstration Base (117° 39′ 19″ E, 28° 59′ 46″ N) in Sizhou Town (Jiangxi, China). The base has a long-standing relationship with our research team; therefore, all plants were collected with permission. We firstly collected the hyperspectral images based on 75 PTRs’ outer surfaces (Fig. 2a). Note that one PTR sometimes grew more than one root simultaneously, and these roots had to be divided because of their large sizes. Therefore, some excessively large plants were divided into several parts, resulting in 120 independent samples in this study. Then, these samples were cut into transverse slices with thicknesses of 4 mm to collect cross-section images (Fig. 2b). In total, 120 outer surface samples and 1350 cross-section samples were obtained (Table 1). The chemical components could have oxidized if the cross-section of the sample was exposed to air for a long time. Thus, cross-section images of each sample were collected immediately after slicing each sample.

**Table 1:** Puerariae Thomsonii Radix (PTR) samples collected in this study.

Growth years	The number of PTRs	The number of outer surface samples	The number of cross-section samples
One year old	24	42	460
Two years old	26	39	437
Three years old	25	39	453
Total	75	120	1350

### Identification of growth years

#### Traditional methods

**Figure 3:**
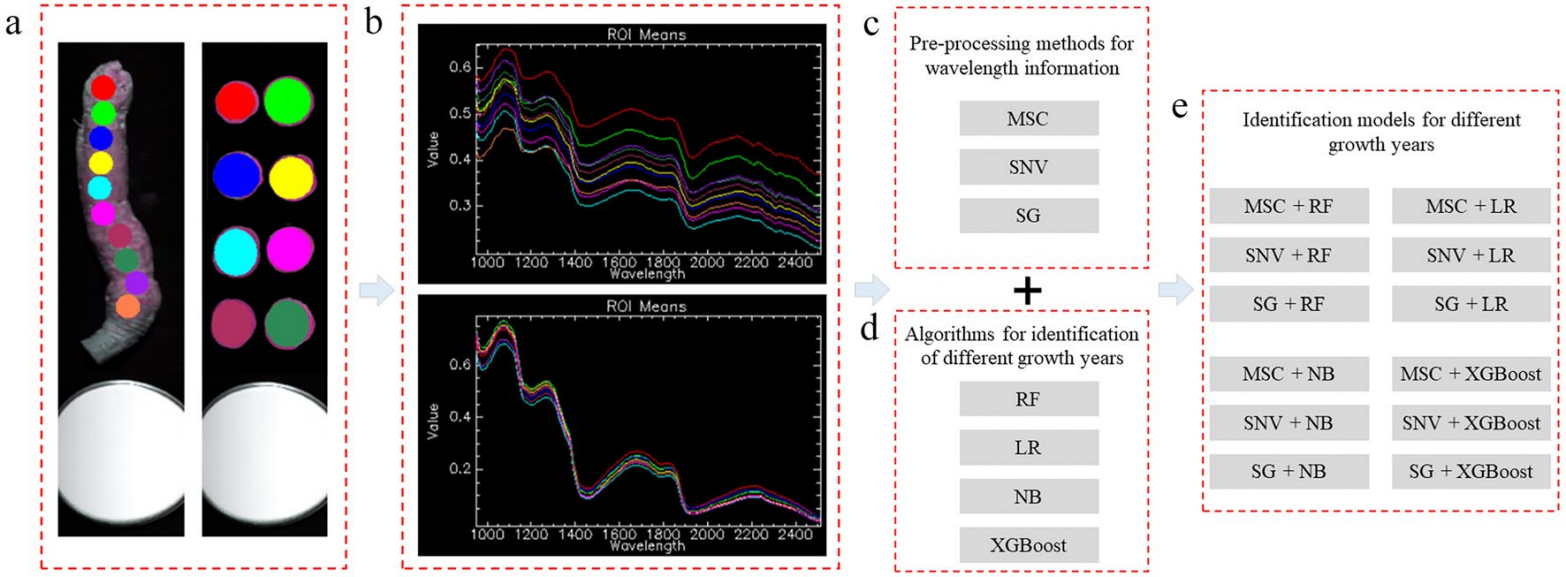
The process of traditional growth year identification methods. *MSC* multiplicative scatter correction, *SNV* standard normal variate, *SG* Savitzky–Golay smoothing, *RF* random forest, *LR* logistic regression, *NB* nave bayesian, *XGBoost* eXtreme gradient boost. The process consists of four steps: selecting ROIs, calculating means, data pre-processing, and building models.

The traditional method for identifying the growth years of PTRs included four steps: (i) selecting several regions of interest (ROIs); (ii) calculating the mean wavelength for each ROI; (iii) pre-processing wavelength information; (iv) identifying growth years based on the calculated wavelength information (Fig. 3).

**Figure 4:**
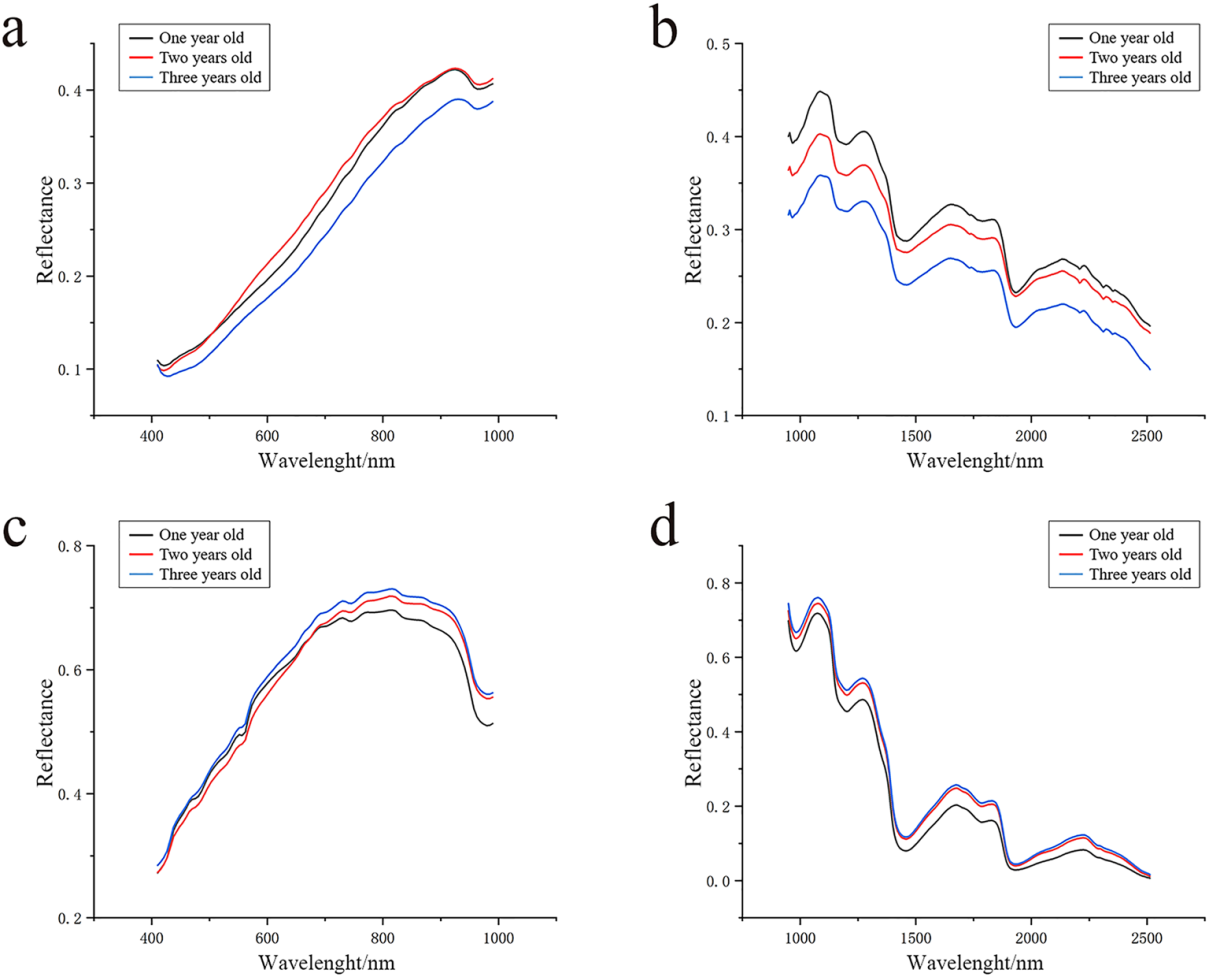
The mean of reflectance for outer surface samples (**a**,**b**) and cross-section samples (**c**,**d**) based on VNIR lens (**a**,**c**) and SWIR lens (**b**,**d**).

The first two steps were performed using the ENVI software (Exelis Visual Information Solutions, Inc., USA). A total of 10 ROIs were randomly selected from each image of the outer surface sample. Therefore, 1200 (= 120 × 10) outer-surface-based ROIs were obtained. Based on the cross-sectional samples, each slice shown in Fig. 2b was taken as a single ROI. Thus, 1350 cross-section ROIs were extracted (Fig. 3a). Then, we calculated the mean wavelength of the extracted ROI using the ENVI software and displayed the mean value in the line charts, as shown in Fig. 3b. We gathered the reflectance of all three growth years and then calculated the mean value of each, as shown in Fig. 4.

The methods used to pre-process the wavelength information included MSC, SNV, and SG smoothing^10,13,20^. MSC can be used to eliminate specular reflection and scatter errors in hyperspectral images and effectively reduce the noise variance in data^21^. It is widely used in multi-wavelength calibration modeling^22^. SNV can remove additive and multiplicative effects in spectra^23^. After SNV processing, the interference of light scattering and baseline shift will be eliminated^24^. SG is a weighted average method that can minimize the loss of valuable information^25^. It can reduce the influence of noise and effectively improve the signal-to-noise ratio of a spectrum^12^. In this study, we used these three most common methods as pretreatment methods. The last step was to classify the growth years of PTRs based on the calculated wavelength information of ROIs using machine learning methods. In this study, random forest (RF), logistic regression (LR), naive Bayesian (NB), and eXtreme gradient boost (XGBoost) were used to predict the growth years of PTR. In this study, we set the number of decision trees to 500 for RF. Additionally, we used two strategies to feed selected ROIs’ information of an image into machine learning models: the mean value of the selected ROIs’ wavelength or all the ROIs’ wavelength were input to each model (i.e., RF, LR, NB, and XGBoost). The baseline model with the higher performance for the two abovementioned strategies was reported and compared with our deep-learning-based models.

#### The proposed method

**Figure 5:**
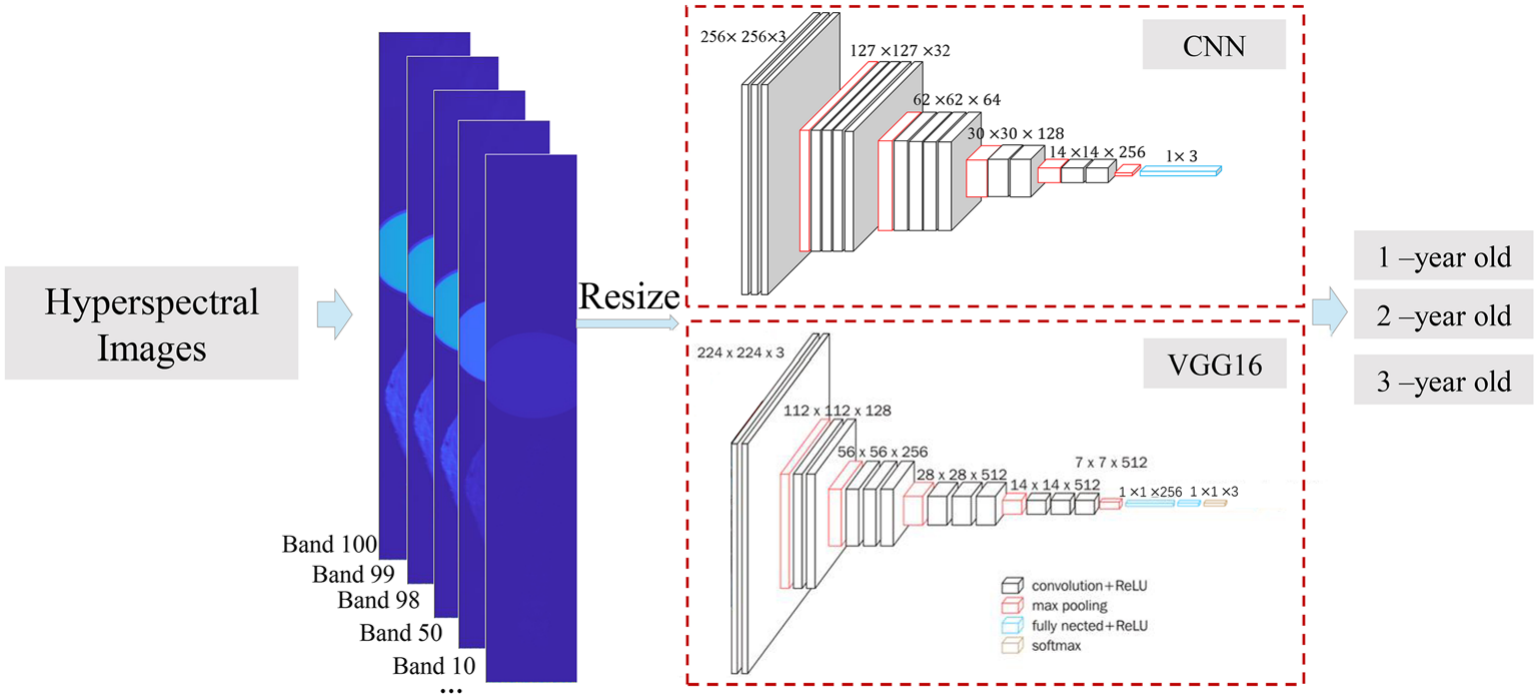
The CNN-based classification framework (VGG16^26^).

The abovementioned traditional method, which requires hand-crafted features, is highly time-consuming and difficult to use when selecting ROIs and calculating wavelengths. Moreover, this process is objective and loses information. Therefore, we proposed a new method based on deep learning without a manual pre-processing step (Fig. 5). The CNN architecture included four layers of convolution, wherein the batch size, the number of epochs, and the learning rate was set to 4, 100, and 0.003, respectively. The CNN was trained with an adaptive moment estimation optimizer with a rectified linear unit (ReLU) activation function. We defined the loss functions as cross-entropy.

In addition to the CNN-based network, we also used VGG16^26^ herein to identify the growth years of PTRs. VGG16 is a special convolutional neural network model, which has a total of 16 layers, including 13 convolution layers and 3 fully connected layers. Compared with other network models, VGG16 adopts a unified 3 × 3 convolution kernel in the whole process. Such a relatively small kernel size is conducive to increasing the depth of network structure, and a large enough number of parameters can be used to learn more complex patterns and achieve better classification effects.

After the sample information is collected by the hyperspectral device, hyperspectral image information is generated. Output of the hyperspectral images from 108 and 288 channels as individual RGB images. The "multibandread" function is a built-in function for reading hyperspectral data in MATLAB software. In this study, we use this function to read hyperspectral data and obtain a single-band image, which is saved as a PNG image with a bit depth of 24. Then, all these images were fed into CNN and VGG16 according to [B, C, H, W] (B: batch size, C: RGB three-channel, H: height, W: width).

The hyperspectral images were firstly divided into a list of two-dimensional images according to the bands. Each VNIR and SWIR lens file contained 108 and 288 bands. Thus, a hyperspectral image was split into 108 and 288 two-dimensional images. We performed five-fold cross-validation and divided the training set and testing set by 7:3 for each band (not merely 459.2 nm). The wavelength of 459.2 nm is an example presented herein. For example, from the 120 outer-surface images achieved in 459.2 nm based on VNIR (the corresponding wavelength was 459.2 nm), 83 (70%) images were used for training, and 37 (30%) images were used for testing purposes. The data for the three different growth years contained 29, 27, and 27 images in the training set and 13, 12, and 12 images in the testing set, respectively (Table 2). The basis for the wavelength selection was the classification results (i.e., F1-score). The wavelengths based on which predictive models showed high performance were selected in this study.

**Table 2:** Training set and testing set for wavelength 459.2 nm (outer surface from VNIR).

	One year old	Two years old	Three years old	Total
Training set	29	27	27	83
Testing set	13	12	12	37
Total	42	39	39	120

Take 459.2 nm as an example to illustrate the data distribution in each dataset.

We trained and tested these methods on a computer (Intel (R) Core (TM) i9-12900K CPU@3.19 GHz, GeForce RTX 3090, 64 GB RAM, Windows 11–64-bit, Python 3.8, PyTorch). During the five-fold cross-validation, the trained model was applied to the test data to quantify model performance. The precision, recall, and F1-score were used as the main metrics to compare models’ performance in this study. The identification of PTRs’ growth year was a ternary classification task, wherein we used the macro-average when calculating the precision, recall, and F1-score.

Generally, the prediction results included positive and negative results, and according to the relationship between the prediction results and the actuality, we obtained combinations of true positive (TP), true negative (TN), false positive (FP), and false negative (FN). Precision refers to how many true positives there are in all the positive prediction results, and recall refers to how many true positives there are in the correct prediction results. The precision and recall are defined as follows:

2 $$Precision=\frac{TP}{TP+FP} \times 100\%,$$

3 $$Recall=\frac{TP}{TP+FN }\times 100\%.$$

F1-score is calculated with precision (P) and recall (R); the closer its value is to 1, the better the prediction result is. The F1-score is defined as follows:

4 $$F1=\frac{2\times P\times R}{P+R}\times 100\%.$$

### Experimental materials

All the plant materials in the manuscript were collected from the Puerariae Cultivation Demonstration Base. This base has a long-term relationship with us, and we have permission to collect Puerariae Thomsonii Radix. In addition, we promise that all procedures were conducted in accordance with the relevant guidelines.

## Results and discussion

### Predictive performance

**Figure 6:**
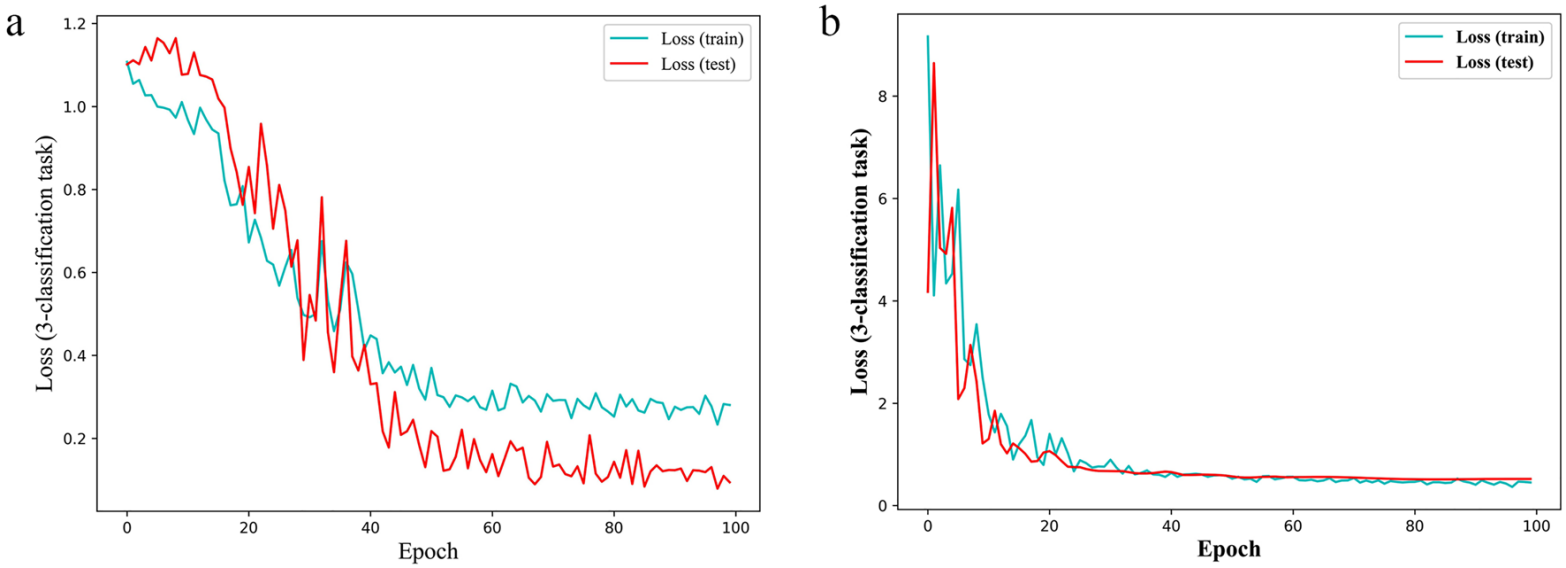
The loss functions of (**a**) CNN and (**b**) VGG16.

The training loss of CNN and VGG16 decreased gradually in the early stage and stayed stable with the increase in the number of training epochs (Fig. 6), indicating that the models had been fully trained. At the end of the 100-epoch training, we achieved 0.2801 and 0.4505 cross-entropy loss for CNN and VGG16, respectively. The fully trained CNN and VGG16 were then estimated using a testing set. Table 3 shows the highest predictive performance of a model trained on images of 108 bands (VNIR lens) and 288 bands (SWIR lens).

**Table 3:** Predictive performance of identifying growth years of PTRs (%).

Model	Outer surface + SWIR			Outer surface + VNIR			Cross-section + SWIR			Cross-section + VNIR		
	Precision	Recall	F1	Precision	Recall	F1	Precision	Recall	F1	Precision	Recall	F1
RF + MSC	61.83	61.97	61.84	64.65	65.17	64.43	70.99	61.90	59.72	66.51	66.67	65.89
RF + SNV	56.84	56.84	56.78	56.34	55.98	54.66	59.85	59.52	58.78	62.75	61.90	61.41
RF + SG	56.84	56.84	56.78	55.56	56.20	55.63	60.41	61.90	59.63	62.75	61.90	61.41
LR + MSC	73.58	55.77	49.37	65.48	53.42	54.08	69.72	54.76	51.18	66.39	61.90	60.43
LR + SNV	54.20	53.63	53.50	62.96	59.83	60.44	67.30	66.67	63.38	68.82	66.67	65.76
LR + SG	65.44	61.75	58.48	61.22	57.05	57.77	64.10	64.29	59.56	66.67	64.29	62.54
NB + MSC	55.57	54.06	54.34	65.17	65.17	65.17	67.30	66.67	63.38	63.59	61.90	60.97
NB + SNV	46.96	45.30	44.38	46.39	45.94	46.11	67.24	66.67	63.69	63.49	61.90	55.57
NB + SG	46.96	45.30	44.38	46.39	45.94	46.11	69.84	69.05	66.34	63.49	61.90	55.57
XGB + MSC	64.68	59.83	59.44	66.35	64.96	64.32	77.28	64.29	62.53	67.97	66.67	66.84
XGB + SNV	50.40	45.94	47.16	50.95	50.64	48.91	66.59	64.29	61.75	64.01	64.29	62.91
XGB + SG	50.40	45.94	47.16	47.17	47.86	46.23	68.25	66.67	64.44	64.01	64.29	62.91
CNN^a^	58.82	60.01	59.33	90.24	90.11	90.15	52.30	51.65	51.83	59.56	59.65	59.60
VGG16^a^	86.90	85.47	84.80	81.15	81.38	81.02	63.62	63.62	63.58	69.94	70.16	69.93

The testing set result of model (out of the 108/288 created models based on 108/288 bands) with the highest performance was presented herein.

^a^Result of five-fold cross-validation.

In Table 2, we took a band as an example to show the sample size. Each band in this study had the same size of sample. The result of the model (out of the 108/288 created models based on 108/288 bands) with the highest performance is presented in Table 3. Under the VNIR lens, the CNN with the F1-score of 90.15% performed best based on the outer surface dataset. Under the SWIR lens, VGG16 with the F1-score of 84.80% performed best based on the outer surface dataset. Among traditional methods, the NB + MSC combination attained the highest F1-score of 65.17% trained on the outer surface dataset under the VNIR lens. In comparison, the highest F1-scores of the deep learning methods for the outer surface and cross-section were 90.15% for the CNN and 69.93% for VGG16, both from the VNIR lens. Notably, the value of 90.15% achieved using the CNN + Outer surface + VNIR combination was also the best F1-score of all, and the improvement in the discrimination accuracy was 38.33% compared with that achieved using the traditional methods.

Features are key to the learning of traditional machine learning models, and there can be a low number of features and a high level of information loss during ROI selection, all these factors will have an impact on the identification results. In contrast, deep learning has the ability of end-to-end learning, which can effectively reduce bias and thus improve accuracy. The results show that it is feasible to establish a deep-learning-based model to identify different growth years of PTRs, especially based on outer surfaces under a VNIR lens.

We then used the CNN and VGG16 that performed best in the ternary classification task to identify whether a PTR could be used in traditional Chinese medicine (i.e., whether a PTR was 1 year old or not). As shown in Table 4, based on the cross-section dataset under the VNIR lens, the F1-scores of the CNN and VGG16 both reached above 88%. Based on the outer surface dataset, the F1-scores of the CNN and VGG16 reached 93.51% and 92.90%. This result demonstrates the feasibility of quality control for PTRs using deep learning algorithms. It is noted that merely the highest predictive performance of a model trained on images of 108 bands (VNIR lens) or 288 bands (SWIR lens) is presented in Table 4.

**Table 4:** Five-fold cross-validation results of testing set of models for predicting 1-year-old PTRs (%).

Dataset	Model	SWIR			VNIR		
		Precision	Recall	F1	Precision	Recall	F1
Outer surface	CNN	94.74	92.31	93.51	92.31	92.31	92.31
Outer surface	VGG16	97.14	87.18	91.89	93.50	92.31	92.90
Cross-section	CNN	77.24	84.82	80.85	86.32	90.99	88.60
Cross-section	VGG16	79.53	90.18	84.51	85.59	90.99	88.21

Additionally, we also found that the performances of models based on the outer surfaces were higher and more robust than that based on cross-section images. The highest F1-score reached 93.51% when growth years were identified based on the outer surface, while the best F1-score was 88.60% based on cross-section images. This result indicated that the growth years of PTRs can be identified using the HSI system on outer surfaces without destroying samples.

In the previous research regarding the identification of age or years, Duan et al.^27^ used six models to identify the ages of cotton seeds, wherein CNN and SVM models achieved satisfactory results, with the identification accuracy being higher than 98%. Wang et al.^28^ proposed an identification method for the identification of the geographical origin and growth years of maize seeds based on the PLSDA model. The accuracy of the testing set reached 98.39%. Bao et al.^29^ established a nonlinear ELM model based on effective wavelengths to classify the different producing years of Dried Tangerine Peel, reaching 93.33% accuracy. It can be seen that HSI technology is effective in the identification of growth years, and the results of this study are consistent with other research. In the domain of the quality control of traditional Chinese medicine, models based on HSI combined with deep learning algorithms were applied to classification and component research, which achieved excellent outcomes^30,31^. The outstanding performances of deep learning algorithms in conjunction with HSI meant they successfully realized the identification of PTRs’ growth years. In the future HSI research, we could attempt to apply deep learning to other aspects of PTR quality control.

### Wavelength selection

There is a large amount of redundant information in full-wavelength data. One of the aims of this research was to find the wavelength based on which the deep-learning-based models identified PTRs with high performance. Then, this selected wavelength could be used in future HSI technology to develop rapid identification equipment for PTR.

**Figure 7:**
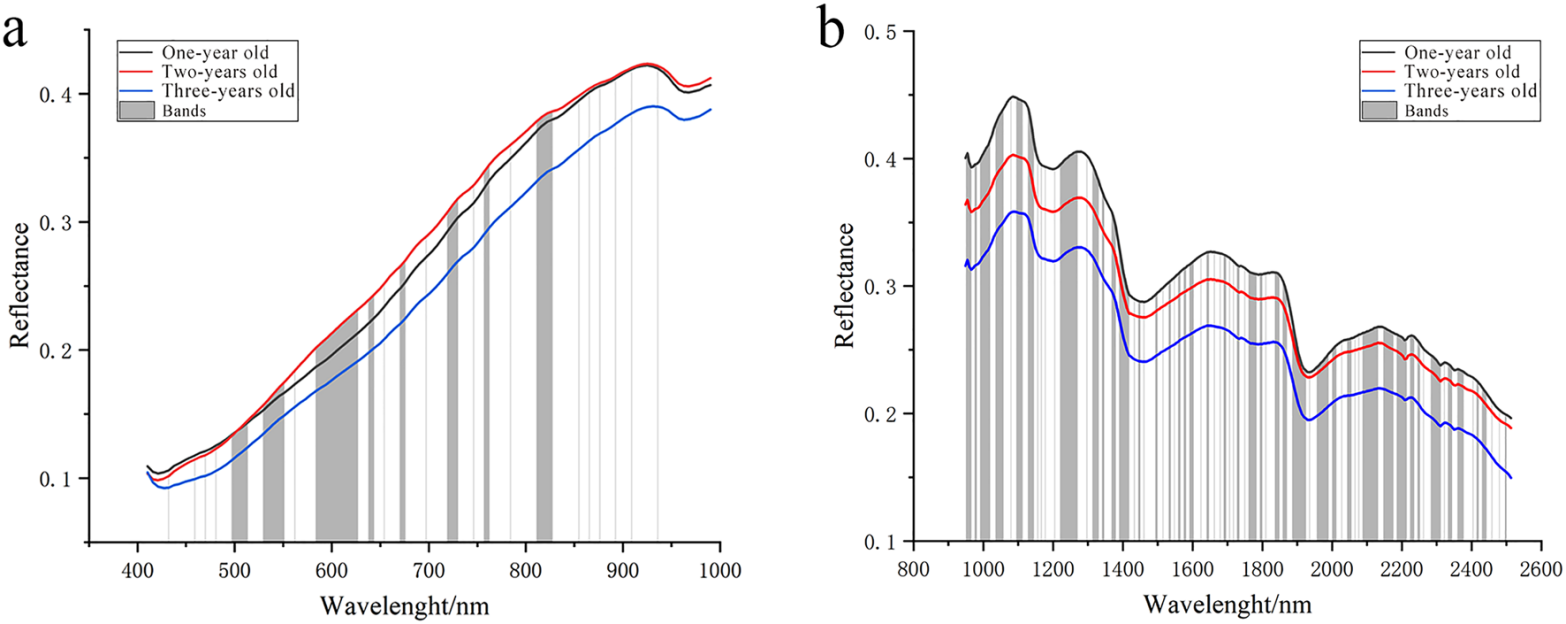
The bands with F1-scores higher than 90% based on the outer surface. (**a**) The selected bands of the binary classification task under VNIR lens; (**b**) the selected bands of the binary classification task using under SWIR lens. In total, 48 and 174 bands were selected under VNIR and SWIR lenses, respectively.

The bands based on images in the CNN and VGG16 that showed an F1-score higher than 90% were all screened out. In the binary classification task that identified whether a PTR was one year old or not, the CNN and VGG16 models simultaneously showed F1-scores higher than 90% based on 48 bands under the VNIR lens and 174 bands under the SWIR lens. The selected bands are shown in Fig. 7. These selected bands can guide the future identification of growth years of PTRs based on the HSI system.

### Characteristic absorption bands

In the effective wavelengths, 540, 605, 1450 and 2371 nm correspond to the characteristic absorption bands of the functional groups of starch. Among them, the wavelengths at 540 nm and 605 nm corresponded to the fourth and fifth overtone regions of -O–H from starch^30^. The wavelength at 1450 nm corresponded to the first octave band spectrum of the fundamental frequency of stretching vibration of –O–H from starch^32^. The wavelength at 2371 nm corresponded to the –C–H second overtone combination and the –CH_2_ overtone combination with deformation vibration from amylose^30^. It was found that during the growth of PTR, the chemical composition accumulated from year to year with a large variation in content, especially in the first few years. In conjunction with the chemical composition corresponding to the effective wavelengths, it is likely that starch is the key chemical component in identifying the growth years of PTRs.

### Accumulation of chemical components

Compared with the prediction results of the cross-section, the accuracy of the outer surface is obviously higher and relatively stable. This may be related to the accumulation of chemical components during the growth of PTR. With the increase of growth time, it will form the fusiform root which is thick in the middle and thin at both ends. This is the storage organ of Pueraria root, which is the part of edible and medicinal value. Similar to other plants of the same genus, the cross section anatomical structure of PTR is mainly periderm, multilayer vascular tissue and secondary xylem from the outside to the inside, each layer of vascular tissue contains phloem and xylem, and the thicker the site, the more layers there are. The researchers^33,34^ confirmed that the periderm the outermost phloem were the main accumulation sites of chemicals, particularly flavonoids, and the content gradually decreases from the outer layer to the inner layer. When the spectrum irradiates the sample, it can not only collect the information on the sample surface, but also penetrate a certain depth to collect the information inside the sample. This is why hyperspectral imaging can be used for nondestructive testing. In the experiments on the penetration depth of nIR spectra of various agricultural products, the researchers found that the maximum penetration depth of nIR spectra of agricultural products was about 2 cm^24,35^, which was consistent with the main accumulation sites of chemical components of PTR. Therefore, HSI technology can be used to identify the growth years of PTR only by collecting surface information of samples, without destroying samples at all.

## Conclusions

In this study, we used a deep-learning-based method in conjunction with HSI technology to identify growth years of PTR. The VNIR and SWIR lenses of HSI equipment were used to collect the information of the outer surface and cross-section of PTR. The CNN model achieved the highest recognition accuracy of 90.15% and 93.51% for both the ternary classification task with different growth years and the binary classification task of “whether a PTR could be used in traditional Chinese medicine”, respectively. Furthermore, the accuracy of the outer surface was generally higher than that of the cross-section, which may be related to the site of Chemical compositions accumulation during the growth of PTR. The results demonstrated that the proposed method is nondestructive, rapid, and effective for the quality control of PTR. Moreover, this method can be easily implemented in the identification of growth years and quality control for other traditional Chinese medicines.

## Data Availability

All data generated or analysed during this study are included in this published article.

## Abbreviations

PTR: Puerariae Thomsonii Radix
PTB: *Pueraria Thomsonii* Benth
HSI: Hyperspectral imaging
MSC: Multiplicative scatter correction
SNV: Standard normal variate
SG: Savitzky–Golay smoothing
RF: Random forest
LR: Logistic regression
NB: Naive bayes
XGB: EXtrem gradient boost
CNN: Convolutional neural networks
VGG16: Visual geometry group network 16
ROI: Regions of interest
HPLC: High performance liquid chromatography
VNIR: Visible and near-infrared lens
SWIR: Short-wave infrared lens
